# The rate of telomere loss is related to maximum lifespan in birds

**DOI:** 10.1098/rstb.2016.0445

**Published:** 2018-01-15

**Authors:** Gianna M. Tricola, Mirre J. P. Simons, Els Atema, Raoul K. Boughton, J. L. Brown, Donald C. Dearborn, G. Divoky, John A. Eimes, Charles E. Huntington, Alexander S. Kitaysky, Frans A. Juola, David B. Lank, Hannah P. Litwa, Ellis G. A. Mulder, Ian C. T. Nisbet, Kazuo Okanoya, Rebecca J. Safran, Stephan J. Schoech, Elizabeth A. Schreiber, Paul M. Thompson, Simon Verhulst, Nathaniel T. Wheelwright, David W. Winkler, Rebecca Young, Carol M. Vleck, Mark F. Haussmann

**Affiliations:** 1 Department of Biology, Bucknell University, Lewisburg, PA 17837, USA; 2 Department of Animal and Plant Sciences, The University of Sheffield, Sheffield S10 2TN, UK; 3 Groningen Institute for Evolutionary Life Sciences, University of Groningen, 9700AB Groningen, Netherlands; 4 Department of Biology, University of Memphis, Memphis, TN 38152, USA; 5 Department of Biological Sciences, University of Albany, Albany, NY 12222, USA; 6 Department of Biology, Bates College, Lewiston, ME 04240, USA; 7 Friends of Cooper Island, Seattle, WA 98112, USA; 8 Department of Biological Sciences, University College, Sungkyunkwan University, Suwon 16419, Korea; 9 Department of Biology, Bowdoin College, Brunswick, ME 04011, USA; 10 Institute of Arctic Biology, University of Alaska, Fairbanks, AK 99775, USA; 11 Department of Biological Sciences, Simon Fraser University, Burnaby, British Columbia, Canada V5A 1S6; 12 ICT Nisbet & Company, Falmouth, MA 02540, USA; 13 Department of Life Sciences, The University of Tokyo, Tokyo 113-8654, Japan; 14 Department of Ecology and Evolutionary Biology, University of Colorado, Boulder, CO 80309, USA; 15 National Museum of Natural History, Smithsonian Institution, Washington, DC 20560, USA; 16 Institute of Biological and Environmental Sciences, University of Aberdeen, Aberdeen, AB24 3FX, UK; 17 Department of Ecology and Evolutionary Biology, Cornell University, Ithaca, NY 14853, USA; 18 Department of Ecology, Evolution, and Organismal Biology, Iowa State University, Ames, IA 50011, USA

**Keywords:** telomeres, bird, lifespan, ageing, senescence, comparative analysis

## Abstract

Telomeres are highly conserved regions of DNA that protect the ends of linear chromosomes. The loss of telomeres can signal an irreversible change to a cell's state, including cellular senescence. Senescent cells no longer divide and can damage nearby healthy cells, thus potentially placing them at the crossroads of cancer and ageing. While the epidemiology, cellular and molecular biology of telomeres are well studied, a newer field exploring telomere biology in the context of ecology and evolution is just emerging. With work to date focusing on how telomere shortening relates to individual mortality, less is known about how telomeres relate to ageing rates across species. Here, we investigated telomere length in cross-sectional samples from 19 bird species to determine how rates of telomere loss relate to interspecific variation in maximum lifespan. We found that bird species with longer lifespans lose fewer telomeric repeats each year compared with species with shorter lifespans. In addition, phylogenetic analysis revealed that the rate of telomere loss is evolutionarily conserved within bird families. This suggests that the physiological causes of telomere shortening, or the ability to maintain telomeres, are features that may be responsible for, or co-evolved with, different lifespans observed across species.

This article is part of the theme issue ‘Understanding diversity in telomere dynamics'.

## 1. Introduction

With advancing age, organisms experience gradual, functional deterioration that leads to diminished performance and a rising risk of mortality. While ageing, or senescence, is a common occurrence across taxonomic groups, the pattern and the pace of the ageing process are variable both within and among species [1]. Understanding the processes that underlie this variation remains a central question across diverse fields of biology. Within evolutionary biology, a better understanding of the underpinnings of the ageing process can ultimately give insight into life-history trade-offs and the evolution of lifespans [2].

One factor that contributes to the ageing phenotype is cellular senescence [3,4]. This process causes an irreversible change to a cell's state, in which the cell ceases to divide and undergoes distinctive phenotypic alterations, including an altered secretory profile that can damage nearby healthy cells [3,5]. Because the number of senescent cells rises during the ageing process, there is a growing loss of the regenerative capacity of cells with age [6]. Cellular senescence occurs as a complex response to excessive extracellular or intracellular stresses, including, but not limited to, severe DNA damage, mitochondrial deterioration, oxidative stress and telomere dysfunction [5]. The progressive erosion of telomeres, the noncoding DNA sequences at the end of linear eukaryotic chromosomes, ultimately triggers a permanent DNA damage response that causes cells to enter senescence [7]. While telomere shortening is only one contributing factor to cellular senescence, it has become a biomarker for senescent cells [8] and the physiological state of an organism [9,10].

The structure of telomeres is generally consistent across eukaryotes, suggesting that telomeres are an ancient and effective guardian of the genome [11]. Consistent with this, telomeres play a broad role in the maintenance of chromosomal genomic stability. Normally, the very end of the telomere folds back on itself to form a structure referred to as the ‘T-loop’, and along with associated proteins, effectively caps the ends of chromosomes. However, as cells replicate, their telomeres shorten due to replication restrictions of DNA polymerase at the ends of chromosomes [12]. Telomere shortening during DNA replication may also be propagated by single-stranded breaks in telomeric regions due to oxidative stress [13]. This progressive telomere shortening will ultimately expose an uncapped free chromosome end that leads to permanent cell cycle arrest [14]. Telomere dysfunction, then, augments the ever-growing pool of senescent cells and could thereby contribute to the decline in tissue function and integrity that is a hallmark of ageing [15].

The ties between telomere loss and cellular senescence suggest an important role of telomere shortening in age-related declines of physiological function. In support of this, a number of human studies have found that individuals with shorter telomeres also have reduced life expectancy [16–19], though other studies do not report this relationship [18,20,21], and see [22] for a discussion of the causal role of telomere shortening in ageing. One particularly interesting within-pair analysis of Swedish twins reported that the twin with the shorter telomere length also had a mortality rate that was three times higher than their co-twin [19]. Consonantly, a growing number of nonhuman studies in natural populations, particularly in wild birds, also show that survival prospects are related to telomere length [23–30]. And a notable report on zebra finches suggests that early life telomere length (at 25 days of age) is a very strong predictor of longevity [30].

While the number of studies exploring links between telomere length and survival within species continues to grow, there have been relatively few comparative studies exploring how telomere biology is associated with ageing rates or lifespans among species. Comparative studies provide a powerful tool to explore vertebrate evolution of ageing in the wild as these studies take advantage of the wealth of variation in lifespans among species generated by long-term selection across different environments. In particular, birds are an especially attractive model for comparative studies of ageing as they are conveniently monitored using ringing and exhibit wide variation of life-history traits [31].

Comparative work has revealed that while absolute telomere length does not seem to relate to longevity among species ([32–35], but see [11]), the rate at which telomeres shorten may offer better insight into the evolution of the lifespan of a species [35]. The first study, done almost 15 years ago, found that species-specific rates of telomere degradation are predictive of maximum lifespan among five avian species and eight mammalian species [35]. To our knowledge, since that time only two additional studies have explored this question, and both supported the original finding, suggesting that variation in telomere degradation rates among species is indicative of distinct levels of telomere maintenance [36,37]. These studies made some advances in experimental design and analysis as Dantzer and Fletcher [36] increased the number of species studied while controlling for phylogeny, and Sudyka *et al.* [37] focused on longitudinal datasets. However, both studies also relied on pre-existing data that were generated by a variety of techniques to measure telomere length; and even within a method, different laboratories can produce widely different results [38,39]. In addition, many of these studies apply the relative qPCR method of telomere measurement. While this technique offers quick and comparable telomere measurements within a study, it is not appropriate for quantitative telomere length comparisons among laboratories or species [39]. Alternatively, the telomere restriction fragment (TRF) assay is more time-consuming but provides an absolute telomere length based on a physical molecular marker that produces more commensurable results across species [39]. Studies based on data that were all generated using the same telomere measurement methodology and analysis while controlling for phylogeny are still sorely needed [9,40].

Here, we explore a number of nonmutually exclusive hypotheses related to telomere length with a comparative dataset, while controlling for phylogeny. The first hypothesis is our own and builds on the work outlined above [35–37]. Furthermore, our comparative dataset allows us to test three other hypotheses regarding telomere biology recently raised in the literature.

(i) Our main hypothesis was that rates of telomere loss are associated with maximum lifespan of species, so that those species with slower rates of telomere erosion also have longer maximum lifespans [35]. Because of the comparative nature of this study, patterns of telomere length and age in a population or species may also be caused by selective disappearance of individuals with short telomeres [41] and we discuss this as well.
(ii) A recent comparative study using phylogenetic analyses of over 60 mammalian species reported that mean telomere length of a species is inversely correlated with lifespan [11]. To our knowledge, this relationship has yet to be tested in a phylogenetically controlled study of birds.
(iii) The telomeric brink hypothesis [42] postulates a causal role for telomere shortening in shaping longevity. If critically short telomeres increase the risk of mortality, then a corollary to this hypothesis is that species with shorter mean telomere lengths and faster telomere loss rates should also have shorter lifespans, which we test here.
(iv) Another recent hypothesis in telomere biology is that long telomeres shorten more quickly than short telomeres, possibly because longer telomeres are more sensitive to telomere-damage events [26]. To our knowledge, this hypothesis has only been evaluated within species, and we tested whether there is any support across species of birds.

## 2. Methods

### (a) Species

**Figure 1.**
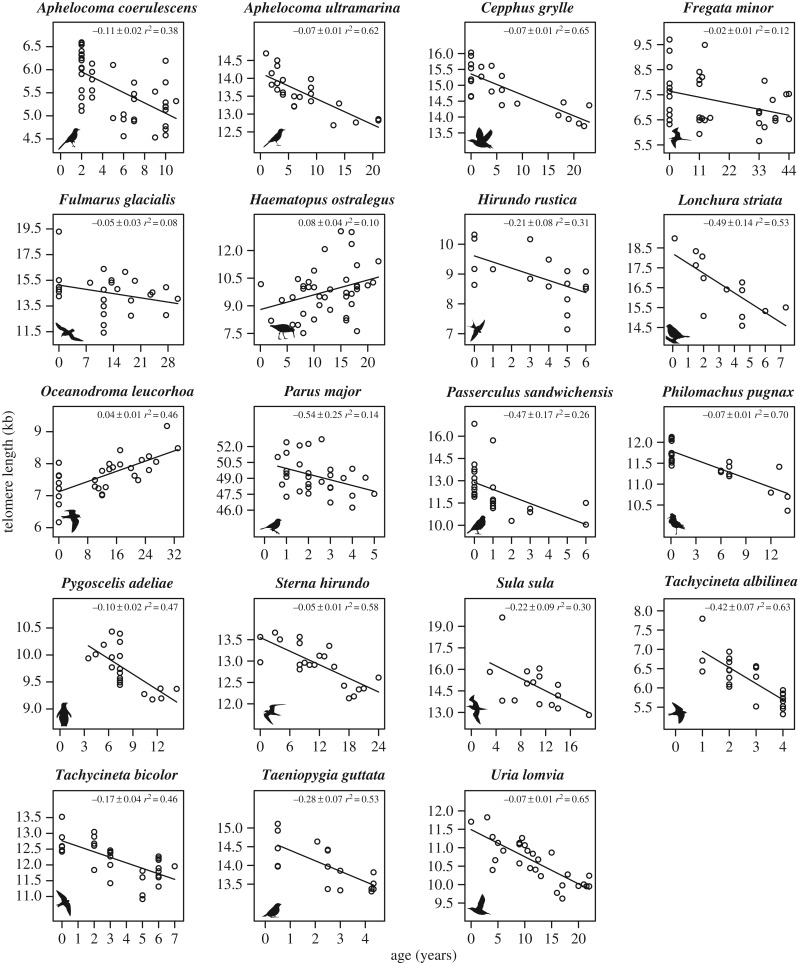
Telomere length (from whole blood measured by TRF analysis) as a function of age in 19 bird species included in the comparative analysis. The lines are linear regressions, and the slope of the regression line for telomere length versus age was used as the telomere rate of change (TROC). The slope of the regression, its standard error and the *r*^2^ are printed within the panel of each species.

We explored telomere shortening in cross-sectional blood samples from 19 avian species representing 5 orders (table 1 and figure 1). We chose species in which long-term study populations were available allowing us to sample individuals over a wide range of their predicted maximum lifespan (table 1). Maximum lifespan estimates for these species range from 7 to 50 years, and were based on natural, long-term study populations. Sex was unknown for a substantial number of individuals in many of the species, and thus, sex was not included in the analysis. We acknowledge that a potential bias in our results may arise from sex differences in mortality as males and females often differ in mortality and lifespan [48] and telomere attrition rates can also differ by sex [49]. Because sex was unknown, the average maximum lifespan between males and females was used in our analyses. While there may be some error in the maximum lifespan estimates, we do not think it would be enough to change our conclusions. Although some studies have suggested that median or mean lifespan may reflect differences in the ageing process more accurately, these values were not available for all species. In addition, maximum lifespan estimates allowed us to be consistent with previous studies [35–37].

**Table 1.** Cross-sectional estimates of blood cell telomere rate of change (TROC) from 19 avian species studied (including order and family), observed maximum lifespan (with literature references) and body mass. Sample size (number of individuals) is included for each species along with the range of ages sampled. *Personal communication from D.W.W., 2011.

species' common name	order (family)	sample size	maximum lifespan (years) [reference]	age range sampled	average adult body mass (g)	TROC (bp yr^−1^)
*Pygoscelis adeliae* Adelie penguin	Sphenisciformes (Spheniscidae)	20	24 [43]	3.5–14.4	4300	−95
*Fulmarus glacialis* Northern fulmar	Procellariiformes (Procellariidae)	27	50 [44]	0–30.5	910	−47
*Oceanodroma leucorhoa* Leach's storm petrel	Procellariiformes (Hydrobatidae)	29	36 [44]	0–33	45	39
*Fregata minor* Great frigatebird	Suliformes (Fregatidae)	35	43 [44]	0–44	1400	−22
*Sula sula* Red-footed booby	Suliformes (Sulidae)	15	23 [44]	3–19	1017	−217
*Haematopus longirostris* Oystercatcher	Charadriiformes (Haematopodidae)	40	43 [44]	0.1–22	660	80
*Calidris pugnax* Common ruff	Charadriiformes (Scolopacidae)	21	14 [45]	0–14	180	−73
*Cepphus grille* Black guillemot	Charadriiformes (Alcidae)	22	29 [44]	0–23	375	−66
*Uria lomvia* Thick-billed murre	Charadriiformes (Alcidae)	28	29 [46]	0–22	1120	−74
*Sterna hirundo* Common tern	Charadriiformes (Laridae)	21	33 [44]	0–24	120	−53
*Aphelocoma coerulescens* Florida scrub-jay	Passeriformes (Corvidae)	43	16 [47]	2–11	80	−112
*Aphelocoma ultramarine* Mexican jay	Passeriformes (Corvidae)	23	25 [44]	1–21	130	−72
*Hirundo rustica* Barn swallow	Passeriformes (Hirundinidae)	17	16 [44]	0–6	18	−205
*Tachycineta albilinea* Mangrove swallow	Passeriformes (Hirundinidae)	26	12*	1–4	15	−418
*Tachycineta bicolor* Tree swallow	Passeriformes (Hirundinidae)	31	12 [44]	0–7	19	−174
*Parus major* Great tit	Passeriformes (Paridae)	30	15 [44]	0.6–4.6	18	−535
*Passerculus sandwichensis* Savannah sparrow	Passeriforme (Emberizidae)s	24	8 [44]	0–6	22	−472
*Taeniopygia guttata* Zebra finch	Passeriformes (Estrildidae)	17	12 [44]	0.5–4.33	13	−279
*Lonchura striata* Bengalese finch	Passeriformes (Estrildidae)	13	7 [44]	0.125–7.33	13	−495

### (b) Laboratory methods

All samples analysed in this study were measured using the TRF assay following previously established methodology [26,50,51]. The TRF assay was developed over 25 years ago and is still widely used to validate other telomere measurement techniques [39]. The assay uses restriction enzyme digestion of genomic DNA followed by Southern hybridization to a radioactive probe containing a terminal repeat to measure mean telomere length from a distribution of TRFs. The traditional TRF assay does have some disadvantages, among them that along with the terminal telomeric repeats, it also measures interstitial telomeres and is biased against the detection of short telomeres. A variant of this, the in-gel TRF assay that we used here, resolves both of these issues by only probing the short G-strand overhang [39,52]. It is important to note that telomere measurements for 17 of the 19 species were measured in a single laboratory (M.F.H.), while the remaining two species (*Haematopus ostralegus* and *Parus major*) were measured with consistent methodology adopted from the aforementioned laboratory. Removing these two species from the analysis does not change any of the following results. In addition to consistency within the TRF technique, blood sampling methods and storage, and DNA extraction are known to influence telomere length estimates [39]. In the light of this we used consistent storage and extraction protocols, and if small changes were made, we were able to validate that, within this sample set, it did not influence telomere measurement. For specific methodology on the in-gel TRF assay used in this study see Haussmann *et al*. [51].

### (c) Telomere length analysis

Gels from all 17 species were imaged on a phosphor screen with a Typhoon Variable Mode Imager (Amersham Biosciences, Buckinghamshire, UK) to visualize telomeres. The amount of radioactive signal (optical density, OD) in each lane corresponds with the amount of telomere at that position on the gel (i), and was quantified by densitometry in ImageJ (v. 1.51). Background signal from nonspecific binding of the radioactive probe was subtracted from all OD measures. The specific molecular markers on each gel differed because gel conditions were optimized based upon a species' particular telomere distribution (1 kb DNA ladder (1–10 kb); 1 kb plus DNA ladder (1–12 kb); Lambda DNA/EcoR1 +HindIII (1–21 kb); 1 kb DNA extension ladder (1–4 0 kb), Invitrogen; *λ* DNA Monocut (2–49 kb) New England Biolabs; DNA Marker XV (2–49 kb), Roche; PFG Marker 1 (15–200 kb), New England Biolabs). However, regardless of the molecular marker used, the distance each band of the molecular marker migrated (i) was plotted against the molecular weight in kilobases and converted into molecular weights (L) using a three-parameter log-linear function. The mean TRF length (called mean telomere length hereafter for simplicity) for each individual was calculated using: mean TRF = ∑(ODi * LI)/∑(ODi), where ODi is the densitometry output at position i, and Li is the length of the DNA (kB) at position I [52].

### (d) Statistical analysis

For each species, we estimated the species-specific telomere rate of change (TROC, bp yr^−1^) as the slope of the linear regression line for telomere length versus age [35]. We did this because while we tried to sample as widely across the age range for each species as possible (table 1), we were limited to what was available. Thus, we made a heuristic assumption of a linear rate of telomere loss across all ages as it allowed for unbiased TROC estimates due to differences in the range of the species lifespan sampled. While longitudinal studies report that telomere loss rate is faster early in life [40], interestingly, we did not observe clear deviations from this assumption (figure 1); but as more telomere comparative data become available for telomere length (TL), nonlinear relationships should be explored. Differences among species' mean telomere length and TROC were assessed in a linear model. The relationships between telomere length or TROC with maximum lifespan among species were assessed using comparative analysis.

In comparative analysis, shared ancestry violates the assumption of independence among data points, and including phylogenetic information statistically accounts for such dependence. To this end, phylogenetically corrected regressions were analysed using generalized least squares assuming a Brownian correlation structure in package ape in R [53]. The phylogeny we used was extracted from a bird supertree [54]. The most parsimonious tree was selected from a Bayesian distribution of a 1000 trees using BEAST [55]. Our models therefore do not incorporate phylogenetic uncertainty [56]. The phylogenetic signal—the amount of variation among species explained by shared ancestry [57]—in both TROC and mean TL was analysed in phytools [58]. Both Pagel's *λ* and Blomberg's K were tested (with only K presented, as both yielded similar results [59]). Trait evolution was plotted on the phylogeny in phytools [58] in R.

Our main hypothesis centred on if and how TROC is associated with maximum lifespan across species (hypothesis i). However, we also explored whether mean telomere length is associated with maximum lifespan across species, as is the case in mammals (hypothesis ii [11]). To this end, we fitted several models around these predictors, and also included body mass as a covariate [60]. Body mass (log_10_-transformed) was included because of the clear associations between body mass and longevity [61]. Mean telomere length and TROC were log_10_-transformed prior to analysis. Telomere length increased with age in two species (*H. ostralegus* and *Oceanodroma leucorhoa*), resulting in a negative TROC, and hence TROC values were log_10_ (*x* + 100)-transformed. Including or removing these covariates allowed us to investigate any sensitivity of our results to mean telomere length and body mass, but the results were similar in all models. We prefer the presentation of full models rather than performing model selection, since full models show the full range of the predictors investigated (both significant and nonsignificant) and are not as likely to inflate type I error [62].

In addition, our comparative dataset allowed us test two other specific hypothesis presented in the literature. First, we considered the telomeric brink hypothesis (hypothesis iii [42]), which suggests that telomere shortening is causal in the ageing process, and when telomeres become too short they cause death. Here, the prediction is that species with both short average telomere length and faster telomere shortening rates would also have shorter maximum lifespans. If such a relationship is present in the data this should result in an interaction between mean TL and TROC against maximum lifespan of a species. Second, we tested the prediction that species with longer telomeres may exhibit faster telomere shortening, which was suggested previously within-species (hypothesis iv [63]). We performed a phylogenetic regression of mean TL against TROC, where a positive relationship would suggest that those species with longer telomeres also show more rapid telomere loss.

## 3. Results

**Figure 2.**
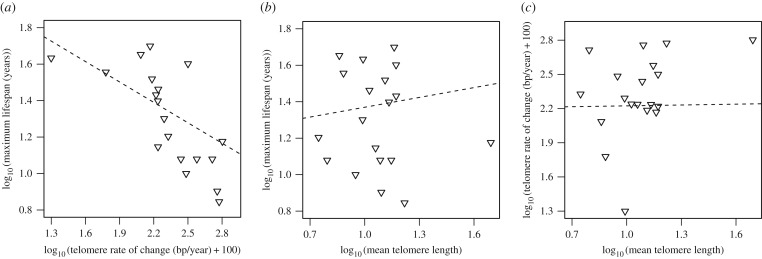
Maximum observed lifespan as a function of (*a*) telomere rate of change (TROC) and (*b*) mean telomere length in 19 bird species. (*c*) Mean telomere length plotted against TROC in 19 bird species. The dashed lines represent the regressions from the phylogenetic regressions without any other covariates included.

Mean telomere length and TROC differed substantially among species (figure 1, linear model: both *p* < 0.0001). Some species show very sharp declines in telomere length with age, while others even increase with age. Interestingly, TROC was strongly associated with maximum lifespan across species, with species of shorter maximum lifespan having greater TROC (figure 2*a*, hypothesis i). This relationship is robust to the inclusion of the different covariates tested (table 2). There was no relationship between mean telomere length of a species and maximum lifespan (table 2 and figure 2*b*, hypothesis ii). Additionally, the interaction between mean telomere length and TROC against species-specific maximum lifespan was not significant (hypothesis iii, interaction: −0.60 ± 0.55 *p* = 0.29, also without the inclusion of body mass, *p* = 0.21), suggesting that TROC is not more determinative of species' maximum lifespan in species with short absolute telomere lengths. A species' TROC was also not associated with the species’ mean telomere length (table 2 and figure 2*c*, hypothesis iv).

**Table 2.** Tests for the first two hypotheses (see §§1,2) in a phylogenetically corrected regression (* indicates *p* < 0.05, ±denotes s.e.). Models were tested with and without body mass (log_10_-transformed) as a covariate, and with and without mean telomere length as a covariate. Telomere rate of change (TROC) is the only significant and strong predictor of maximum lifespan variation among species, with greater telomere loss rates associating with shorter maximum lifespan (hypothesis i). TROC was not related to mean telomere length of a species (hypothesis ii).

	without body mass		body mass included		
dependent	TROC (+100, log_10_)	mean TL (log_10_)	TROC (+100, log_10_)	mean TL (log_10_)	body mass (log_10_)
hypothesis i Max lifespan (log_10_)	−0.37 ± 0.14*		−0.34 ± 0.14*		0.11 ± 0.07
		0.18 ± 0.16		0.18 ± 0.16	0.13 ± 0.08
	−0.38 ± 0.14*	0.19 ± 0.14	−0.35 ± 0.14*	0.19 ± 0.14	0.11 ± 0.07
hypothesis ii TROC (+100, log_10_)		0.02 ± 0.24		0.03 ± 0.25	−0.07 ± 0.13

**Figure 3.**
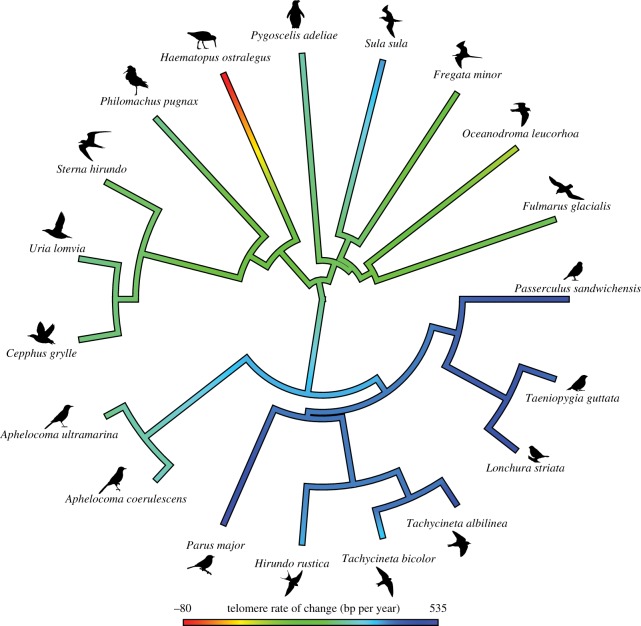
Trait evolution of telomere rate of change (TROC) mapped to the phylogeny in 19 bird species. Colours indicate different levels of the trait value (transformed values were used for mapping, but linear values are depicted for illustrative purposes in the legend). TROC shows a strong phylogenetic signal and the major families or clades of species which were included in this analysis show similar rates of telomere loss with age.

A phylogenetic signal was detected for TROC (*K* = 1.21, *p* < 0.001; figure 3), but not for absolute telomere length (*K* = 0.35, *p* = 0.60). Note that when *K* is larger than 1 it indicates that phylogenetically related species are more similar than expected under Brownian motion [59].

## 4. Discussion

Our study confirms previous reports that species with greater TROC have shorter maximum lifespans (hypothesis i [35–37]). By contrast, among the species sampled here, mean telomere length was not associated with longevity (hypothesis ii). Because TROC so accurately explains maximum lifespan (table 2, figure 2), the physiological causes of telomere shortening, or the ability to maintain telomeres, could be partially responsible for the different lifespans observed across species. In support of this suggestion, TROC shows a strong phylogenetic signal, whereas absolute telomere length does not, although it does vary widely among species. TROC, in contrast to absolute telomere length, therefore, appears evolutionarily conserved and selected within bird families. We acknowledge that this inference is less firm when phylogenies are small, but the difference in phylogenetic signal is striking when considering the potential biological significance of telomere length loss compared to absolute telomere length.

The pattern reported here between TROC and lifespan may be caused in part by selective disappearance, in which certain phenotypes are preferentially removed from a population [41]. Since our study is cross-sectional in nature due to the lack of long-term study populations and the long lifespans of some species, the relationship between TROC and lifespan could be a result of selective loss of short-lived individuals from the environment. This selective disappearance of particular individuals [41,64]—those with short telomeres—can cloud the relationship between telomere loss and age in a cross-sectional context [65]. For example, the positive relationship between telomere length and age seen in Leach's storm petrels (*O. leucorhoa*) is most likely due to the longest-lived individuals starting with the longest telomeres and variation in telomere length decreasing with age owing to the selective disappearance of individuals with short telomeres [65]. It is also possible that selective disappearance is responsible for the positive relationship seen in the Eurasian oystercatcher (*H. ostralegus*). It is difficult to translate a cross-sectional pattern within species to within-individual processes for that species. Future longitudinal studies will allow us to distinguish between population differences that result from the removal of certain phenotypes earlier in life than others. At the moment, longitudinal studies of ageing in free-living populations are rare, but are needed because of their greater power to identify age-related changes compared to cross-sectional studies [31,66,67]. However, in a comparative context, cross-sectional studies can still broadly inform us about the biology of ageing and lifespan, though we lose the ability to firmly conclude that these patterns are resulting from processes within individuals.

The degree to which selective disappearance differs among species can be caused by differences in extrinsic mortality rates and differences in how age-related mortality is influenced by telomere biology. Classical evolutionary theories of ageing predict that extrinsic mortality levels should be inversely correlated with evolved lifespan [68]. In other words, short-lived species generally face higher risk of death due to predation, starvation or accident. Given this, one interpretation of our results is that if there is selective disappearance of individuals with short telomeres, this pattern may be partially concealed in populations of species where individuals are removed from the population due to random processes regardless of their condition. This is not to say that selective disappearance based on telomere length is not occurring in short-lived species, only that it may be more readily obscured. Conversely, if telomere erosion does in fact increase mortality risks, then one might expect that this would be more evident in long-lived species with lower rates of extrinsic mortality where functional senescence is more easily observed. If extrinsic mortality differences are a major driver of selective disappearance based on telomere length this may explain why the species displaying patterns that most closely resemble selective disappearance are two of the longest-lived species we studied, the storm petrel and oystercatcher.

However, regardless of species differences in extrinsic mortality, the degree to which telomere biology affects intrinsic ageing processes may also differ across species, thereby affecting selective disappearance. In other words, some species might have a stronger relationship between telomere biology and survival prospects compared to others. Hence, selective disappearance could be stronger in species that are long-lived and for whom telomere biology is more important. Therefore, selective disappearance may be due to both differential association of intrinsic mortality with telomere length and differences in extrinsic mortality that reduce the importance of telomere length in determining mortality at the population level. Regardless of either of these causes, the comparative pattern we find suggests that as the longevity of a species increases, telomere biology becomes increasingly important. Moving forward, more longitudinal data are sorely needed to disentangle the possible scenarios outlined above that can result in the cross-sectional relationship we report here. Such efforts will allow us to understand more details of the deteriorative process of senescence in general [67], and how selective disappearance occurs in species of differing lifespan in particular.

While selective disappearance may be partially responsible for the pattern we observe between TROC and lifespan, another possibility is that telomere erosion is a potential mechanism underlying the evolution of lifespan in birds, with short-lived birds losing more telomeres each year compared to long-lived birds. A recent meta-analysis of 14 avian species reported that the rate of telomere loss is correlated with maximum lifespan estimated from a composite measure of life-history traits [36]. Another recent study, using existing data from longitudinal studies in bird species, confirmed a negative relationship between the rate of telomere shortening and maximum longevity [37]. Both of these recent studies provide additional support for the hypothesis that telomere attrition is correlated with interspecific rates of ageing. The underlying mechanisms responsible for this relationship are still unknown, and selective disappearance, physiological mechanisms that ameliorate telomere loss, or some combination of the two may be at play.

In search for physiological mechanisms that underlie the evolution of lifespan, comparative analyses have revealed that across avian and mammalian species, those species with longer lifespans also have cells that are both more resistant to external stressors [69] and have lower rates of mitochondrial free radical generation [70]. One possible physiological explanation for the different rates of telomere loss in the avian species in our study is that they also had different levels of oxidative stress. Oxidative stress can increase single-stranded breaks in telomeric regions of DNA that can cause telomere shortening during DNA replication due to a proposed pausing of the replication fork [13], though this work is mainly based on *in vitro* evidence, and whether it holds *in vivo* has recently been questioned [71]. Nevertheless, species with higher levels of oxidative stress may experience more rapid telomere shortening. Interestingly, this may be due in part to free radicals' preferential damage to the guanine-rich telomeric sequence in comparison to other regions of DNA [72]. This may allow telomeres to act as a free radical magnet, soaking up damaging free radicals while protecting coding regions of the chromosome. Accordingly, if long-lived species experience lower levels of oxidative stress, then their telomeres may have less exposure to the damaging attack of free radicals.

Another mechanism that could underlie the relationship between the rate of telomere loss and maximum lifespan of species is differential levels of telomerase expression. In mammals, a phylogenetically controlled comparison in rodents found that telomerase activity relates to body mass [33]. Another comparative mammalian study confirmed the relationship between telomerase activity and body mass and also found an inverse relationship between mean telomere length and lifespan [11]. The study authors suggest that short telomeres along with telomerase suppression are necessary for the evolution of large body size and longevity in mammals, apparently as a cancer suppression mechanism. Interestingly, while mean telomere length varies by an order of magnitude in the species we studied (5–50 kb), we do not find a relationship between mean telomere length and lifespan (hypothesis ii). The lack of a phylogenetic signal may suggest that telomere length evolves rapidly within a lineage, though this scenario is unlikely as the analysis includes some closely related species (*Aphelocoma* and *Tachycineta* species pairs, for example). Another possibility is that telomere shortening is more important on specific chromosomes, but that selection is neutral for other chromosomes. Every chromosome end contributes to the mean telomere length of a species with the in-gel TRF assay, and methods such as the single-telomere length analysis could better determine the role that telomere length on single chromosome ends might play in lifespan.

Previous studies in birds actually showed higher levels of telomerase expression in cells of species with longer maximum lifespans [73]. This suggests that longer-lived avian species may have evolved mechanisms that promote telomere maintenance through telomerase expression. Compared to mammals as a whole, birds have reduced body mass and relatively long lives. And the idea was proposed that a smaller body size and fewer cells may allow birds to have higher telomerase activity and longer telomeres without the associated high cancer risk [40]. While this idea is intriguing, more phylogenetically controlled work on interspecific variation in telomerase expression as well as comparative experimental work on telomerase activity in tumour cells is critically needed [74,75].

The direct role of telomeres in organismal ageing is still unclear [22]. While telomere shortening may directly contribute to senescence [76], there also may be no causal relationship between telomere biology and ageing [22,77]. Recently, the telomeric brink hypothesis (hypothesis iii [42]) postulated a causal role for telomere shortening in shaping longevity. Specifically, the authors note that individuals who are born with short telomeres also have shorter telomeres in adulthood, which results in a greater degree of cellular senescence and mortality, potentially through atherosclerosis. We tested this hypothesis in our comparative study of birds, but did not find a stronger relationship between TROC and maximum lifespan for species with short average telomere lengths. Given that atherosclerosis is relatively common in avian species [78], this raises the possibility that telomeres may not play a causal role in ageing, but rather serve as a biomarker of other ageing-related physiology [18,69]. However, data exploring telomere loss over an individual's lifespan within a species are necessary to better evaluate this hypothesis. Another recent hypothesis in telomere biology is that long telomeres shorten more quickly than short telomeres, possibly because longer telomeres are more sensitive to telomere-damage events or even random DNA damage (hypothesis iv [26,79]). To our knowledge this has only been evaluated within species, but across species we did not find support for this hypothesis. But, while we might expect species with longer average telomere length to lose telomeres more rapidly we cannot account for differences in telomere maintenance mechanisms among species, which could cloud these relationships.

## 5. Conclusion

Identifying the evolutionary importance of how different physiological mechanisms cause the ageing process can be aided by comparative studies. The results of this study clearly show that rates of telomere loss are strongly associated with species longevity in birds. In addition, this relationship is evolutionarily conserved and selected within bird families. Avian species that are better able to maintain their telomeres or conversely for which telomeres are more strongly associated with survival, causing selective disappearance, may experience lower rates of cellular and organismal ageing. While this study highlights the connection between telomere biology and the pace of life among species, we need to continue to uncover how within-individual processes that affect ageing in an ever-changing environmental backdrop relate to telomere loss.

## Data accessibility

Data are available within figures and tables in the manuscript.
